# Lean Adoption in Healthcare: Between Promise and Practice

**DOI:** 10.3390/healthcare14152403

**Published:** 2026-08-05

**Authors:** Jarosław Garski, Dariusz Walkowiak

**Affiliations:** Department of Organization and Management in Health Care, Poznan University of Medical Sciences, Poznań 61-701, Poland

**Keywords:** lean adoption, lean healthcare, implementation barriers, sustainability, organizational change, performance measurement, continuous improvement

## Abstract

Background: Lean Management (LM) is recognized for its significant potential to improve efficiency and quality in healthcare. However, despite its promise, widespread and sustainable adoption across healthcare organizations remains paradoxically limited, with many initiatives failing to move beyond localized, short-term successes. This study examines the systemic barriers that hinder the long-term integration of LM in healthcare settings, to understand why a gap persists between its theoretical potential and practical reality. Methods: A systematic literature review was conducted following the PRISMA 2020 guidelines. A search of Scopus, Emerald Insight, PubMed/MEDLINE databases and Google Scholar was performed to identify peer-reviewed articles published in English between 2015 and 2025. The review focused on studies addressing the adoption, implementation, or sustainability of LM in healthcare. Data on measurement frameworks, influencing factors, and reported barriers were extracted and synthesized to develop an explanatory model. Results: The synthesis of the ten included studies yields three thematic clusters, each documented in the reviewed evidence and, in this analysis, connected as mutually reinforcing loops: (1) the Measurement–Accountability Gap, (2) the Tools–Philosophy Disconnect, and (3) the Complexity–Resistance Cycle. The proposed connections among these clusters are elaborated in the Discussion. Conclusions: The principal contribution is the synthesis of these previously reported barriers into an integrated conceptual model in which they are connected as mutually reinforcing mechanisms rather than treated as independent factors, offering healthcare leaders a structured perspective for interpreting the pattern of limited sustained adoption.

## 1. Introduction

### 1.1. Healthcare Cost Crisis and Need for Efficiency

Healthcare costs are rising dramatically worldwide. In the US, healthcare spending grew by 7.5 percent in 2023, reaching $4.9 trillion [1], while in Europe, projections suggest unsustainable increases in government expenditures, reaching 27.4% of GDP by 2070 [2]. This increase in costs is driven partly by the growing availability of effective but costly medical treatments and technologies [3]. The global population is aging, requiring greater healthcare resources. Projections indicate that healthcare spending will increase significantly due to more older adults around the world [4]. Additionally, medical advancements may also exacerbate organizational challenges stemming from inefficiencies in the healthcare system [5]. The search for operational efficiency in healthcare is becoming a necessity to ensure sustainable and accessible medical services.

Healthcare organizations face challenges in keeping up with the cost of providing medical services [6]. Finding ways to improve operational efficiency and quality of care has become a central concern. One potential solution from the automotive industry is Lean Management (LM). The basic premise of LM is that greater efficiency can be achieved through continuous improvement aimed at eliminating waste and maximizing value-adding activities [7]. Over the past decade, LM has gained significant traction within the healthcare sector, with numerous studies indicating its potential to improve operational effectiveness [8]. It has become one of the most widely implemented management doctrines in some countries, such as Finnish health and social care [9].

### 1.2. The Paradox of Limited Adoption

However, despite these promising advancements, empirical studies show that adoption is still highly localized, with small successes. Most transformations focus on implementing one or two LM tools that primarily target patient waiting times, and there is limited evidence of sustainability [8]. Furthermore, some research critically views LM in healthcare, highlighting paradoxical and potentially harmful implementations [10]. Paradoxical, meaning contradictory implementations with unexpected or unintended negative consequences, despite their positive intentions. These criticisms point out that, despite claims of effectiveness, the methodology may produce micromanaging, worker stress, and questionable results. For example, the Virginia Mason Institute failed a safety inspection even though it was praised as a model of efficiency [11]. Such paradoxical outcomes typically occur when organizations focus exclusively on process optimization without adequately addressing the cultural and philosophical foundations necessary for a sustainable Lean transformation, leading to superficial implementations that may actually increase rather than reduce operational complexity [12]. A study analyzing Lean implementation in US hospitals found an unexpected negative association between the extent of Lean education and the screening for clinical conditions, despite the expectation that Lean principles would improve such preventative measures [13].

Documented instances of sustained Lean adoption in specific healthcare systems exist. Rosa and colleagues [14], for example, describe a multi-year, cross-organizational Lean deployment across an Italian regional hospital network, an exception that this review’s paradox framing seeks to explain. The persistent pattern across the broader evidence base, however, is one of localized short-term gains rather than system-wide sustained transformation, and it is this pattern that the present synthesis addresses.

These contrasting perspectives raise the question: *Why does the adoption of Lean management remain limited in healthcare settings despite its potential to improve efficiency?* This research paper investigates the active and continuous adoption of LM principles, tools, and methodologies in healthcare organizations’ daily operations and processes. By examining the methods used to measure adoption, factors that influence it, and the current state of LM adoption rates in healthcare, we aim to understand the complex factors that may limit widespread and sustainable LM adoption in healthcare settings.

### 1.3. Research Significance and Gaps

LM transformation requires significant effort. An organization’s transformation to a lean organization requires a long-term strategic vision, commitment from management, and a culture of change throughout the organization [15]. Adoption, as used in this study, refers to the active and continuous integration of LM principles, tools, and methodologies into a healthcare organization’s day-to-day operations and processes. Three related terms are used throughout this review with distinct meanings: ‘Lean Management’ (LM) denotes the broader managerial system; ‘Lean adoption’ denotes the organizational integration of that system as defined above; and ‘Lean implementation’ denotes the operational deployment of specific Lean tools and practices, which may occur without constituting adoption in the sense used here. Consequently, studies conceptualizing adoption as an isolated implementation event, or those failing to empirically measure sustained integration over time, were excluded. Methodologically, such approaches measure a transient intervention rather than the construct of organizational adoption. True adoption means a systematic commitment to employing it as an overall strategy to meet organizational objectives. Recent reviews and empirical analyses, including [16,17,18,19], typically present barriers to sustained Lean adoption as static lists or taxonomies rather than as interacting mechanisms, an approach consistent with the current state of the evidence base but leaving the dynamics among barriers largely unexamined. Most studies on Lean healthcare barriers adopt a reductionist approach, cataloging impediments as static, isolated events. The specific contribution of this synthesis is the structural integration of these previously reported barriers into a single closed-loop conceptual model that connects mechanisms typically treated as independent in earlier analyses.

Existing studies provide valuable insights into implementation challenges, but appear to leave gaps in understanding why sustained adoption often fails. Table 1 outlines the focus of selected publications to illustrate why this paper’s research question remains open, explicitly demarcating between studies retained for full systematic synthesis and the baseline literature utilized strictly for contextual background.

When discussing long-term adoption, improper implementation of LM can cause several negative consequences. These include an overemphasis on cost reduction at the expense of quality care [13], reduced staff engagement and increased workload leading to burnout and medical errors [21], and a negative impact on the patient–physician relationship [13]. Additionally, no clear link may exist between LM implementation and improving the quality of care, which might worsen some practices [13]. Prioritizing worker wellness and patient value is paramount. LM implementation must be adapted to the specific needs of the healthcare environment and monitored frequently for its impact on quality of care and patient satisfaction.

### 1.4. Measurement Frameworks in Healthcare Lean Adoption

These frameworks are examined in detail because measurement is the operational lens through which Lean adoption is defined, tracked, and evaluated in one of the three barrier domains identified in the Results (Measurement–Accountability Gap). Their strengths and limitations, therefore, directly inform the analysis of why adoption remains uneven despite documented improvement efforts. In healthcare, LM adoption refers to the active and sustained integration of Lean principles, tools, and methodologies into a healthcare organization’s daily operations and processes [22]. It is not simply about using a few Lean tools; it involves a cultural shift towards continuous improvement and patient-centric care. Successful Lean adoption appears to require adapting Lean principles to the healthcare context and ensuring that staff and the organization are ready for implementation to ensure long-term sustainability [19].

#### 1.4.1. How Do We Measure Lean Adoption in Healthcare?

Measuring Lean adoption in healthcare settings remains difficult. Several frameworks have emerged to help organizations provide systematic methods for measuring progress and identifying improvement areas. While detailed data on their popularity and usage levels is limited, some have gained more popularity than others. These frameworks tend to be adaptable, backed by evidence of performance, and aligned with the mission-driven nature of healthcare. Organizations appear to choose frameworks based on their needs, whether cultural transformation, tool-based effectiveness, or integrated excellence. While many other assessment tools and frameworks exist (such as LESAT [23] or the key factors influence [24]), here are three notable frameworks with numerous healthcare applications [13,25]. Other frameworks are less specific to healthcare and do not adequately address its unique challenges. They may lack essential standards due to insufficient validation. Additionally, they can be impractical because of high costs and limited flexibility, making the following three options preferable for healthcare organizations [26].

1. The Healthcare Lean Assessment (HLA) framework is based on the Shingo Prize model [27], a well-established framework for operational excellence. It includes four key dimensions: cultural enablers, continuous improvement, enterprise alignment, and results (quality, cost/productivity, delivery, customer/patient satisfaction, and morale) [28].
2. The Lean Healthcare Implementation Self-Assessment Instrument (LHISI) is a survey-based instrument designed to assess the maturity of Lean implementation across different units within a hospital. It focuses on key Lean principles and avoids the limitations of traditional assessment methods, such as excessive length and reliance on external consultants [29]. LHISI has been validated in Finnish healthcare, demonstrating its feasibility and validity [30]. A study at Helsinki University Hospital employed structured interviews based on the LHISI framework to evaluate Lean maturity and identify factors that support and hinder its implementation [25].
3. The National Survey of Lean (NSL) focuses on three key dimensions: the maturity of Lean adoption, the implementation approach, and Lean performance [31]. It assesses leadership commitment, daily management system use, and staff training in Lean principles [13]. The NSL’s popularity appears to come from its approach to evaluating Lean implementation and its impact on hospital performance [32]. It allows for benchmarking and identifies key factors associated with successful Lean initiatives, making it potentially useful for healthcare organizations seeking to improve quality and efficiency [13]. Table 2 summarizes the strengths and weaknesses of these three frameworks.

While the HLA, LHISI, and NSL are valuable tools for assessing Lean implementation in healthcare, they all have drawbacks. The HLA’s comprehensiveness can overwhelm small organizations, while the LHISI’s focus on specific practices can overlook cultural factors. The NSL, based on self-reported data, is subject to bias. The variety of Lean applications across healthcare organizations, where different departments adopt distinct frameworks and methodologies, complicates the evaluation process. To better understand the bigger picture, we need to understand the unique context and objectives of each unit and the organization as a whole.

#### 1.4.2. What Is Influencing Lean Adoption in Healthcare?

**Figure 1:**
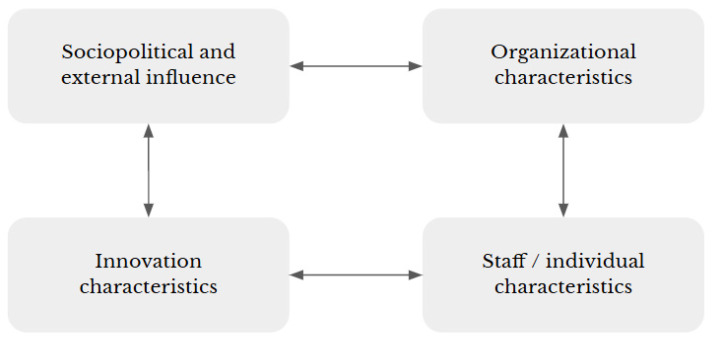
Descriptive conceptual map of factor categories influencing Lean adoption in healthcare as reported across the framework literature.

The practical and sustained adoption of LM in healthcare is a complex process influenced by many interacting factors [19]. Successful adoption goes beyond initial enthusiasm and requires an understanding of the forces contributing to long-term success. Factors affecting adoption can be broadly categorized. First, sociopolitical and external influences create the backdrop for Lean initiatives. Government regulations and policies, collaborative relationships with other healthcare institutions, and the overall economic climate significantly impact the feasibility and prioritization of Lean. Second, internal organizational characteristics play a crucial role. Strong leadership commitment is important for driving change and overcoming resistance [35]. A culture that embraces continuous improvement and innovation and empowers employees is important [18]. Adequate financial, human, and technological resources are also necessary for proper implementation and training [16]. Third, the perceived characteristics of Lean itself influence its acceptance. The complexity of Lean methodologies, the anticipated benefits, and the compatibility with existing workflows affect adoption rates [17,35]. Fourth, individual employee characteristics are highly influential. Positive attitudes towards change, good understanding of Lean principles through adequate training, and high levels of motivation and engagement are essential for successful Lean adoption [16,36]. Additionally, contextual factors such as trust, maturity, and dissemination can significantly influence Lean adoption [15]. Despite identifying these factors, their long-term impact on LM adoption is an often overlooked aspect. The dynamic nature of healthcare makes it difficult to understand whether these factors hinder or help sustain Lean adoption over time. These factors interact with one another across various organizational levels, creating complex interdependencies and feedback loops. Figure 1 presents the category-level relationships reported across these sources at a descriptive level; empirical analysis of the mechanisms connecting these categories was not performed in the present review and remains a direction for future work.

This a priori theoretical framework maps the broader literature to establish a pre-analytic context for the review. This four-category conceptual structure integrates scoping dimensions from the secondary literature, including Wisdom et al. [35] and theoretical construct models from Wagner et al. [19], Kunnen et al. [16], Melo et al. [17], and Hung et al. [18]. While several of these sources (such as Wagner et al. and Melo et al.) were properly excluded from the formal systematic synthesis due to lacking primary, longitudinal empirical adoption data (as detailed in the PRISMA flow), they are retained strictly in this background section to inform the sensitizing framework. This methodological distinction ensures that the theoretical lens remains broad, while the primary empirical synthesis mapped in the Results section (Table 4) is derived exclusively from the ten systematically included studies.

Next, in the results section, we will explore LM adoption in healthcare. While there are various ways to evaluate adoption levels, the research reveals some inconsistencies and gaps. These inconsistencies and gaps are central to our findings, emphasizing areas that need further research and standardized measurements in lean adoption studies.

## 2. Methods

To gain a deeper understanding of the adoption of LM in healthcare settings, a systematic literature review was conducted. This review adhered to the PRISMA 2020 guidelines, ensuring a transparent and reproducible process [37]. The primary objective was to synthesize existing research and identify key areas for future investigation. The review was not prospectively registered with PROSPERO or another systematic review registry. The protocol was developed before the search and documented internally; its principal elements, including the research question, databases, search terms, inclusion and exclusion criteria, and screening procedure, are reported in full in Methods and Supplementary S1. The authors acknowledge that the lack of prospective registration is a procedural limitation: it restricts independent, timestamped verification of the pre-specified methodology and prevents readers from tracking amendments between the initial plan and the final synthesis.

A search was conducted across four sources: three primary academic databases (Scopus, Emerald Insight, PubMed/MEDLINE) and Google Scholar as a pre-specified supplementary source. Google Scholar was retained for three reasons. First, the evidence base for sustained Lean adoption in healthcare is small and dispersed across health services, management, and industrial engineering journals, with uneven indexing coverage, so reliance on primary databases alone carries a nontrivial risk of missing eligible peer-reviewed work published in regional or not yet indexed journals. Second, this risk proved material: one of the ten included studies ([38], IN-06) was published in a journal not indexed in Scopus, Emerald Insight, or PubMed/MEDLINE at the time of the search and was retrievable among the searched sources only through Google Scholar; its omission would have removed the only non-Western study from the synthesis (see Limitations). Third, Google Scholar permitted screening of gray literature and early-stage publications to assess publication bias; records of this type were excluded under the peer-review criterion (Supplementary S1), confirming that identical eligibility rules were applied to all sources consistent with the recommended supplementary role of Google Scholar in systematic reviews [39]. Its known limitations regarding strict search reproducibility are acknowledged; the exact query, interface filters, and retrieval count are documented in Supplementary S1. All records retrieved from Google Scholar were subject to the same peer-review inclusion criterion (Supplementary S1) and the same design-and-scope criteria applied to records from the three primary databases; no separate eligibility track existed.

The search strategy employed a combination of keywords related to both the topic and the setting. Topic-related keywords included “lean adoption,” “lean adoption rate,” “lean implementation,” and “lean sustainability,” while setting-related keywords included “health care,” “healthcare,” and “hospital.” This dual approach aimed to capture a broad spectrum of relevant studies. The search was conducted in November 2025. The complete Boolean search strings used for each of the four databases, including field restrictions, year limits (2015–2025), document-type filters, and language filters, are provided in Supplementary S1, in accordance with PRISMA 2020 [37]. The 2015–2025 publication window applied to the systematic search and screening process, capturing the decade during which healthcare-focused Lean sustainability research shifted from case-report style to systematic empirical assessment and during which two of the three frameworks examined in this review, LHISI and NSL, were formally introduced and validated in the peer-reviewed literature [29,32]. As with other foundational theoretical and instrument-development sources cited throughout this manuscript (e.g., Liker 2004 [40]; Mazzocato et al. 2010 [41]), the original HLA framework [28] predates this window and is cited here as conceptual background rather than as part of the systematically searched evidence base.

The study selection process involved a rigorous two-stage screening. Initially, titles and abstracts were reviewed to identify potentially relevant studies. Those deemed relevant progressed to the second stage, where full-text articles were assessed against predetermined inclusion and exclusion criteria. Title/abstract screening and full-text eligibility assessment were conducted independently by both authors against the criteria specified in Supplementary S1. Disagreements at either stage were resolved through discussion between the authors, with the pre-specified inclusion and exclusion criteria as the reference frame; no third reviewer was required. Formal inter-rater agreement statistics were not calculated for the screening stages; this is acknowledged as a procedural limitation, although all eligibility decisions were criterion-driven and auditable against the pre-specified criteria in Supplementary S1. Studies were included if they were peer-reviewed articles published in English between 2015 and 2025, focused on healthcare settings (such as hospitals and clinics), addressed LM adoption, implementation, or sustainability, and employed qualitative, quantitative, or mixed methods. The requirement for primary empirical data operationalizing adoption rates or utilizing structured measurement frameworks served as the primary methodological filter. Consequently, theoretical conceptualisations of barriers and studies reporting isolated implementation events without longitudinal operationalization of adoption were excluded, as they lacked the empirical basis required to address the specific construct of sustained organizational integration. Furthermore, while fundamental instrument development and psychometric validation studies (such as those establishing the LHISI or NSL frameworks) were utilized to inform the theoretical background, they were formally excluded from the systematic synthesis if they focused solely on instrument properties rather than reporting applied empirical data on adoption rates or implementation outcomes. The same boundary was applied to multiple reports drawing on a single underlying survey program: the descriptive and performance-focused analyses of the 2017 U.S. National Survey of Lean [32,34] and the Finnish national Lean survey [9], whose instruments and samples are carried forward in the included studies IN-03 [31], IN-10 [13], and IN-09 [42], are cited as conceptual background, preventing duplicate representation of overlapping datasets within the ten-study evidence base. Non-peer-reviewed articles, studies not focused on healthcare settings, studies not directly addressing LM adoption or long-term implementation, and review articles, editorials, or opinion pieces were excluded.

The methodological quality of the included studies was appraised using the Mixed Methods Appraisal Tool (MMAT), version 2018 [43]. The MMAT is designed to evaluate qualitative, quantitative descriptive, quantitative non-randomized, quantitative randomized, and mixed-methods studies within a single systematic review, and is therefore appropriate for the design heterogeneity of the ten studies included in this synthesis, as documented in Supplementary S1. Each study was evaluated against the two screening questions and the five design-specific MMAT criteria corresponding to its study design. The evaluation was conducted independently by both authors, and disagreements were resolved through discussion. Overall, MMAT evaluation followed the original instrument’s reporting convention. The results of the appraisal are reported in Table 6.

**Figure 2:**
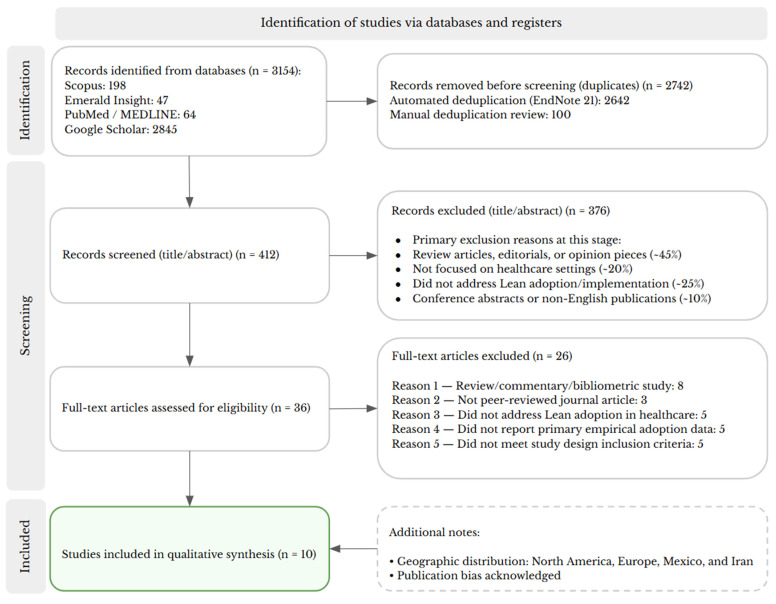
PRISMA 2020 flow diagram of the systematic review. Own elaboration.

The systematic search identified 3154 records across the four databases (Scopus: 198; Emerald Insight: 47; PubMed/MEDLINE: 64; Google Scholar: 2845). After removal of 2742 duplicates (2642 identified automatically using EndNote 21 and 100 identified manually), 412 records were screened at the title and abstract level, 36 proceeded to full-text assessment, 26 were excluded with reasons, and ten studies met all inclusion criteria and were retained for qualitative synthesis. The full PRISMA 2020 flow is shown in Figure 2 and in Supplementary S1. A PRISMA flow diagram (Figure 2) visually represents the study selection process. This diagram illustrates the number of records identified, screened, and included at each stage, providing transparency and clarity.

These ten retained studies represent the entirety of the eligible evidence identified by the systematic search strategy, rather than a purposive or convenience sample drawn from a broader pool of qualifying literature. Data were extracted from the included studies using a standardized data extraction form. This form captures information such as data collection year(s), overall percentage of lean adoption, measurement methodologies, country of origin, research methodology, main barriers to long-term stability, publication date, research summary, and type of healthcare organization. The extracted data were then synthesized using a narrative synthesis approach, allowing for the identification and discussion of key themes and findings. Narrative synthesis was chosen as the appropriate method given the design heterogeneity of the included studies (qualitative, quantitative, and mixed-methods) and the incompatibility of their outcome measures, units, and instruments for meta-analytic pooling. This choice is consistent with established practice for systematic reviews of heterogeneous implementation research, including Kunnen et al. [16] and Wagner et al. [19].

### Data Bias Acknowledgment

There is the risk of bias in the selected data. The research employs various methods conducted across diverse cultural environments and health systems (from global surveys to individual hospital case studies, e.g., the USA, Spain, Finland, Italy, Poland, Mexico, and Iran) and analyzes different dimensions of Lean implementation. This heterogeneity, while intended to inform the research question, can introduce variability into the outcomes. Publication bias, where studies reporting positive results of Lean adoption are published more often than those with negative or inconclusive findings, is also a concern. The included studies span various methods and data-collection approaches, to select sources with information on the prevalence of LM adoption and measurement approaches.

## 3. Results

This study examined three Lean assessment frameworks, emphasizing their core principles, sustainability approaches, and practical applications in healthcare. HLA evaluates overall Lean implementation maturity, LHISI monitors implementation progress, and NSL assesses the impact of Lean on hospital-wide performance. The frameworks present distinct approaches to sustainability, as summarized in Table 3. However, none of these frameworks independently provides the standardized global approach necessary for measuring long-term sustained adoption across various healthcare settings.

Mapping the empirical findings of the ten included studies onto the theoretical framework established in the Background, we categorized the identified factors influencing LM adoption in healthcare into the four predefined areas: sociopolitical/external influences (e.g., government, policies), organizational characteristics (e.g., leadership, culture), innovation characteristics (e.g., complexity, compatibility), and staff characteristics (e.g., attitudes, skills). Each category includes specific factors that the synthesized empirical data show affect the successful long-term adoption of LM (see Table 4).

### 3.1. What Is the Level of Adoption of Lean Management?

The reviewed studies utilize various methodologies to assess LM adoption in healthcare, reflecting different perspectives on what constitutes “adoption” and how it can be measured. A primary distinction lies between qualitative and quantitative approaches, with some studies employing mixed methods to capture a fuller picture [25,44].

Qualitative studies, such as the one by Kabirinaeini et al. (2023), rely on interviews and focus groups with healthcare professionals and experts to understand the practical implementation of Lean [38]. These studies often use coding and content analysis of interview data to identify themes and patterns related to Lean adoption [38]. For instance, the study at Helsinki University Hospital utilized semi-structured interviews with 15 healthcare leaders across three sectors [25]. This approach provides a deep understanding of the experiences and perspectives of those involved in Lean initiatives [25]. Another study used a qualitative approach to analyze the implementation of Lean in several hospitals in the Apulia region of Italy, relying on semi-structured interviews over the years [14]. This study identified three key elements of Lean implementation: introduction, dissemination, and strategic implementation [14]. Most of the analyzed empirical studies [25,42,45,46] focus on the identification of barriers and enablers of Lean adoption. These studies also aim to understand the impact of Lean on staff well-being and patient satisfaction [14,25].

Quantitative studies employ surveys and statistical analysis to measure Lean adoption. The National Survey of Lean (NSL) used in the study by Marsilio et al. (2022) measures Lean adoption across three dimensions: maturity of adoption, implementation approach, and perceived outcomes [31]. This study collected data from public hospitals in the US and Italy, with 282 and 91 responses, respectively [31]. The NSL uses composite indices to assess different aspects of Lean implementation [31]. The Lean Healthcare Implementation Self-Assessment Instrument (LHISI) is another quantitative approach using a survey with a reduced set of 25 items across five dimensions, including leadership, commitment, standard work, communication, and daily management system [25]. The LHISI survey was sent to over 26,000 employees at Helsinki University Hospital with a response rate of 27% [25]. Quantitative measures of Lean include process-related data such as patient wait times, length of stay, and costs [13,14]. The study by Prado-Prado et al. measured and sought to reduce waste in healthcare processes by using the scientific action research approach [45]. They used a methodology involving training and staff participation, detecting waste, and establishing control mechanisms linked to objectives using key performance indicators [45]. Another study conducted in a Polish hospital used value stream mapping and the 5 Whys method to identify waste areas and streamline the patient admission process [46]. The results indicated reduced patient admission process waste and time spent on documentation using the electronic version [46]. The study also reported savings in nursing and medical staff positions [46].

The reviewed studies vary in their definitions of Lean adoption. Some focus on the extent of implementation, such as the number of units using Lean, the use of Lean tools, and the daily management system [31]. Others see adoption as a cultural shift that involves a mindset of continuous improvement and a commitment to eliminate waste [14,25,44,45]. Some quantitative studies operationalize adoption through indices that reflect the degree of organizational support and engagement with Lean principles [31].

Different studies employ varying metrics. Qualitative studies often focus on subjective experiences and perceptions, while quantitative studies use objective metrics related to processes and outcomes. Both types of studies aim to identify factors that either facilitate or hinder the adoption and sustainability of Lean in healthcare settings [25,45,46].

Table 5 summarizes various methods for measuring lean adoption and the challenges associated with sustaining that adoption.

### 3.2. Several Inconsistencies and Gaps Were Identified

- A lack of studies that investigate the long-term sustainability of Lean adoption [14,16,17,45]. Many studies focus on the early stages of Lean implementation, typically analyzing periods of less than 24 months [45]. The need for studies that assess longer-term adoption and outcomes is evident [14]. Looking more broadly, the pharmaceutical and healthcare-related industries do not seem promising. The limited focus on the long-term sustainability of Lean also poses challenges for organizations seeking to maximize the benefits of Lean [47].
- There is a lack of standardized metrics to measure Lean adoption, making it difficult to compare findings across different studies and healthcare systems [16,31,45,46].
- Many studies focus on hospital settings, while other healthcare environments, such as health centers or primary care, may require different methods for measuring Lean adoption [18,45]. Recent evidence suggests that implementing Lean in primary care settings faces unique challenges, including workflow variability and patient–provider relationship dynamics, that differ from hospital environments [12].
- Focus tends toward implementing tools rather than adoption of Lean as a management system and philosophy [16,18,20,25].

Synthesizing these extracted empirical gaps, operational inconsistencies, and reported challenges across the ten included studies reveals three distinct but interconnected areas of systemic friction: (1) a persistent reliance on cross-sectional rather than longitudinal metrics, creating a Measurement–Accountability Gap; (2) an organizational tendency to prioritize discrete operational interventions over foundational cultural change, resulting in a Tools–Philosophy Disconnect; and (3) the friction generated when linear Lean methodologies clash with highly autonomous clinical workflows, initiating a Complexity–Resistance Cycle. These three emergent thematic clusters represent the synthesized output of the reviewed literature and form the basis for the integrated theoretical model elaborated in the Discussion.

### 3.3. Methodological Quality of the Included Studies

The MMAT 2018 evaluation of the ten studies (Table 6) showed moderate-to-high methodological quality. All met the screening criteria. Three studies (IN-01, IN-02, IN-04) satisfied all design-specific criteria, two (IN-06, IN-10) met four, and the rest (IN-03, IN-05, IN-07, IN-08, IN-09) had a mix of “Yes” and “Can’t tell” responses. No study was excluded due to quality. “Can’t tell” responses primarily concerned sample representativeness and nonresponse bias in quantitative descriptive studies (IN-03, IN-05, IN-08, IN-09), hospital sample representativeness (IN-10), and mixed-methods integration depth (IN-07). These reflect common practical constraints in healthcare research, such as limited access and resources.

These quality results informed the interpretation of findings regarding the three barriers and were addressed in the study limitations.

Table 7 maps the ten included studies to the loop(s) each is cited to support in the Discussion, constructed directly from the explicit in-text citations that underlie each loop rather than from a post hoc thematic classification. By detailing the specific ‘nature of contribution’ for each study, this table explicitly demonstrates which empirical results led to the identification of the three mechanisms and highlights the systemic interrelationships synthesized from the extracted data. This citation-based approach was adopted to ensure that the proposed loop structure remains traceable to the specific empirical contributions of the synthesized literature, rather than resting on an inferred or retrospectively assigned thematic fit. Because the citation pattern identified in the Discussion is not mutually exclusive, one study is retained under both loops for which it is cited, rather than being allocated exclusively to a single “principal” domain; this reflects the underlying evidentiary structure of the synthesis rather than an inconsistency in the mapping process.

The MMAT ratings (Table 6) were integrated into the interpretation of the evidence supporting each proposed loop. The ‘Can’t tell’ ratings, which primarily concerned sample representativeness and non-response bias in quantitative descriptive studies (IN-03, IN-05, IN-08, IN-09) and integration depth in the mixed-methods study (IN-07), are distributed across the synthesis rather than concentrated within any single domain. Consequently, no individual loop’s evidentiary base is disproportionately weakened, as each draws on at least one study evaluated without ‘Can’t tell’ flags across most criteria (Table 7). Given the qualitative–narrative nature of this synthesis and the small number of included studies, a formal quantitative sensitivity analysis was neither feasible nor methodologically indicated. The citation-based mapping in Table 7 serves as a transparent alternative: each loop is independently supported by at least one study rated without ‘Can’t tell’ flags, and the flags themselves concern reporting of sample representativeness and non-response rather than the substance of the findings cited as evidence. Evidentiary confidence should therefore be viewed as variable across the component loops rather than uniform.

## 4. Discussion

The integrated model developed in this synthesis moves beyond the independent barriers identified in the prior literature by revealing their underlying systemic interdependencies. For instance, the Measurement–Accountability Gap functions as an operational mechanism of what Leite et al. [48] term ‘ostensible barriers’ surface-level obstacles that mask deeper systemic measurement failures. The Tools–Philosophy Disconnect draws on the tools-versus-transformation distinction documented by Mazzocato et al. [41] and on the culture-determines-sustainability finding of Pakdil and Leonard [49]. The Complexity–Resistance Cycle is consistent with the organizational context dynamics documented by Harrison et al. [50] and the cultural sustainability framework of Willis et al. [51]. The contribution of the present analysis is to synthesize these prior observations into an integrated, self-reinforcing structure derived from the convergent themes of the ten included studies. The methodological evaluation of these ten studies, summarized in Table 6, yielded a moderate-to-high quality profile, with no study excluded on quality grounds; the convergence of findings across studies of differing design categories therefore reflects substantive thematic agreement rather than the dominance of any single methodological approach.

The specific theoretical originality of this review lies not in identifying the individual barriers, each anchored in prior work [41,48,49,50,51], but in integrating them into a single, connected model whose behavior is more than the sum of its components. Unlike previously published models that primarily catalog implementation barriers as static, independent variables, our synthesis proposes an integrated, causal-loop framework. By shifting from a linear descriptive presentation to this dynamic framework, this model untangles how these known elements actively feed into and amplify one another, offering a novel systemic explanation for the widespread failure of sustained adoption. It is the connection between these barriers, rather than their individual identification, that this review contributes; the proposed model draws on prior work that treats the same barriers separately and integrates them into a single interpretive frame.

Our analysis reveals a critical methodological limitation within the included studies utilizing the HLA, LHISI, and NSL instruments [13,25,31]: these frameworks are predominantly applied in cross-sectional research designs rather than for longitudinal tracking. Consequently, the synthesized literature lacks the continuous measurement necessary to empirically operationalize sustained Lean adoption, such as enduring shifts in organizational culture or persistent efficiency gains [28,30]. Furthermore, the measurement landscape across the included studies is fragmented across disparate foci—from economic process outcomes [44] and operational waste [46] to national-culture correlates [42], each employing distinct, non-comparable metrics that preclude integrated longitudinal assessment and compound the accountability gap. This measurement inadequacy may create what Leite et al. (2020) identify as “ostensible barriers”—surface-level obstacles that mask deeper systemic issues [48]. Healthcare leaders are operating under intense financial pressure. They cannot justify continued investment in Lean initiatives without reliable metrics that demonstrate sustained return on investment. The absence of standardized, comparable metrics across healthcare settings means that even successful implementations cannot be effectively benchmarked or replicated. This situation may perpetuate localized and unsustainable efforts.

Second, the Tools–Philosophy Disconnect undermines transformational change. The recurring implementation divergence observed across distinct healthcare systems [14,25,45] fundamentally corroborates and extends Mazzocato et al.’s (2010) diagnosis of organizations limiting themselves to ‘adopting specific lean tools’ rather than undergoing systematic transformation [41]. This phenomenon may occur because tools provide tangible, immediate outcomes that satisfy short-term performance pressures, masking the neglect of the underlying continuous improvement philosophy.

However, this tool-centric approach may not address the cultural and systemic changes necessary for sustained improvement. As Pakdil and Leonard (2015) demonstrate, organizational culture fundamentally determines whether Lean processes can be implemented and sustained [49]. Without the philosophical foundation of continuous improvement and employee empowerment, tool-based implementations remain vulnerable to reversal when leadership changes or competing priorities emerge. As diagnosed by Radnor et al. (2012), healthcare organizations frequently fall into the trap of implementing rapid improvement events while neglecting the underlying philosophy, leading to an “unfilled promise” [52]. This aligns with Liker’s foundational definition of Lean as a deeply ingrained, systemic management culture, rather than a mere toolkit [40].

Third, the Complexity–Resistance Cycle is an implementation barrier driven by the interplay of the four factors in our model: sociopolitical, organizational, innovation, and individual characteristics. This resistance cycle does not merely reflect generic change aversion among staff [16,38], but rather represents an active clash within the complex clinical hierarchy, directly supporting and extending Harrison et al.’s (2016) theories on organizational context dynamics [50]. In healthcare, interacting clinical hierarchies and highly autonomous professional identities create structurally challenging environments for systemic change. When a Lean innovation is introduced, it can increase the perceived complexity for individuals. This often triggers resistance among staff, a sociopolitical dynamic that leadership may interpret as implementation failure. In response, organizational support and resource allocation for essential training are usually reduced. Lack of support compounds implementation challenges, paradoxically increasing both the perceived complexity and staff resistance. This, in turn, may lock the organization in a self-perpetuating cycle.

The role of digital technology illustrates the innovation factor’s impact. Poorly aligned technology can act as the primary catalyst, adding a layer of complexity that initiates the cycle. In contrast, well-integrated digital tools, such as robotic process automation, can simplify workflows, reduce complexity, and support Lean initiatives [53]. Therefore, breaking this cycle requires a holistic approach that addresses these interconnected factors simultaneously. Willis et al. (2016) emphasize this point in their work on sustaining cultural change in health systems [51]. This resistance is particularly pronounced among highly autonomous medical professionals, as standardization efforts associated with “white-collar Lean” often clash with deeply rooted professional identities and established clinical autonomy [54].

The integrated explanatory model: These three barriers are theorized to operate as a self-perpetuating cycle where limited measurement capabilities prevent demonstration of value, leading to continued emphasis on short-term tool implementation rather than long-term philosophical transformation, which increases perceived complexity and resistance, further undermining measurement and sustainability efforts. This model may explain why, despite significant investment and initial enthusiasm, Lean adoption in healthcare remains “paradoxically limited” with “questionable long-term sustainability” [8,24,33].

### 4.1. Practical Implications

Understanding this integrated barrier model has direct implications for healthcare organizations seeking successful Lean adoption. Breaking the cycle requires simultaneous intervention across all three barrier areas, avoiding sequential or isolated approaches.

The results suggest that the successful adoption of LM requires an effort on several fronts. Leadership commitment is essential, requiring leaders to actively support and visibly participate in Lean initiatives rather than simply endorsing them. This leadership role appears essential, as it should result in tangible resource allocation and the strategic integration of Lean into organizational goals; this recommendation is framed by the organizational-readiness constructs of Wisdom et al. [35], used throughout this review as background theory rather than as one of the ten systematically included studies, and is consistent with the leadership-engagement findings reported in the included studies by Rosa et al. [14] and Kunnen et al. [16]. Additionally, empowering staff through training programs and fostering a culture of ownership may help in overcoming inherent resistance to change and drawing on frontline staff knowledge, consistent with findings from Kunnen et al. [16] and the staff-resistance dynamics documented in Kabirinaeini et al. [38].

For measurement systems, organizations must invest in developing hybrid measurement approaches that combine elements from existing frameworks (HLA, LHISI, NSL) with longitudinal tracking capabilities that extend beyond typical 24-month implementation periods. This requires establishing baseline measurements before implementation and committing to multi-year data collection that can demonstrate both process improvements and cultural transformation, consistent with the multi-site benchmarking evidence in Marsilio et al. [31] and the process-outcome analysis in Tierney et al. [13].

Context-specific implementation strategies are critical for the success of LM adoption in healthcare settings. An overly rigid approach to Lean implementation may not be effective. Instead, a phased, adaptive rollout allows for iterative learning and early success demonstrations, which may encourage broader organizational buy-in [15]. Furthermore, fostering a supportive environment by actively connecting with regulatory bodies, professional associations, and collaborative networks may help address sociopolitical barriers and encourage broader adoption of LM [35]. For philosophy integration, organizations must embed cultural assessment and development into the very early stages of tool implementation, preventing culture from being treated as an afterthought. While early “quick wins” are pragmatic and necessary to build momentum, they must be paired with immediate investment in leadership development programs that emphasize continuous improvement mindsets, and establishing cross-functional improvement teams that include frontline staff, consistent with the barrier/facilitator framing of Kunnen et al. [16]. Creating formal mechanisms for employee suggestions and implementation demonstrates genuine empowerment. Digital Lean implementation, while identified as a possible additional implication, is not directly supported by any of the ten included studies and is therefore not proposed as an evidence-anchored implication here.

Standardized metrics appear important for promoting sustained LM adoption. The absence of consistent measurement frameworks impedes benchmarking and the demonstration of long-term value. Implementing metrics is essential for effectively evaluating LM. A well-designed measurement system should cover key performance indicators across various areas, including process efficiency metrics, patient-centered outcome measures, and staff-related outcomes [28]. Assessing the adoption of Lean principles within the organizational culture appears essential. This data collection enables tracking progress over time and illustrates LM’s impact on key organizational variables [9].

### 4.2. Limitations

The rigorous selection funnel (retaining only ten studies from 412 screened records) highlights a profound scarcity of primary empirical research measuring sustained Lean adoption. While this scarcity underscores the paper’s central claim regarding the lack of longitudinal validation, the strict operational definition of “sustained adoption” applied in this review constitutes a limitation, as it inherently excluded studies of localized or cross-sectional Lean implementations that may hold practical clinical significance. Consequently, this strict filtering could influence the generalizability of the proposed barrier model to healthcare organizations in earlier or more fragmented stages of adoption.

The research is limited by the heterogeneity of the selected studies, which differ in methodology, healthcare systems, organizational types, and criteria for defining Lean adoption. This variation makes it challenging to draw firm conclusions, suggesting that a future systematic review with a qualitative meta-synthesis approach may be necessary to better integrate findings.

Additionally, while restricting the review to English-language publications was a practical necessity, it introduced the risk of publication bias. This limitation might exclude important work conducted in other languages, potentially distorting the overall understanding of Lean adoption in healthcare. In addition, the search strategy required one of four exact phrases (‘lean adoption’, ‘lean adoption rate’, ‘lean implementation’, ‘lean sustainability’) in the searched fields; empirical studies describing related phenomena under adjacent labels (e.g., ‘Lean maturity’, ‘transformational performance improvement’) may therefore not have been retrieved, a recognized trade-off between reproducibility and search sensitivity. To mitigate this bias, future research could explore strategies such as searching for gray literature (e.g., conference proceedings, theses, and unofficial reports) and actively engaging with researchers in the field to uncover unpublished or ongoing studies.

Quality evaluation utilized the MMAT instrument, acknowledging the limitations of using a single tool across diverse study designs. The inclusion of only ten studies from 3154 records highlights the scarcity of primary Lean research meeting modern methodological standards, justifying the call for harmonized measurement frameworks. The proposed three-barrier integrated model is a theoretical contribution that requires independent empirical validation before its interactions can be generalized beyond this synthesis.

Finally, generalizability is constrained by geographic concentration: eight of the ten included studies originate exclusively from North America or Europe, one additionally covers Mexico alongside Finland, and one originates from Iran, reflecting the concentration of the peer-reviewed Lean-in-healthcare literature over the 2015–2025 window rather than a search-strategy restriction. The applicability of the proposed three-loop model to low- and middle-income countries, differently financed or organized health systems, and culturally distinct clinical settings, therefore, remains an open question; the single non-Western study included was incorporated into the Complexity–Resistance Cycle rather than analyzed as a distinct data point, and offers limited evidence toward such generalizability on its own. The model is offered as a synthesis of the evidence base as it currently exists, not as a claim about how these dynamics would unfold elsewhere. Language barriers further limited the identification of relevant studies from other regions; future research would benefit from collaborations with researchers in underrepresented regions to develop a more global perspective.

## 5. Conclusions

The narrative synthesis of the ten included studies suggests that limited and unsustained LM adoption in healthcare may be explained by three interconnected barriers that may constitute a self-reinforcing cycle, which we label the Measurement–Accountability Gap, the Tools–Philosophy Disconnect, and the Complexity–Resistance Cycle, together constituting a proposed integrated theoretical account of the adoption gap. The analysis is consistent with the interpretation that limited adoption may not simply reflect implementation challenges but may instead reflect systemic features of how Lean transformation is approached organizationally. This review contributes a synthesis of barriers to sustained Lean adoption that have been previously reported individually, connecting them into a single conceptual model that may support the design of implementation strategies addressing the barriers together rather than in isolation. Healthcare organizations may benefit from understanding why previous Lean implementations have struggled and how to achieve more sustained transformation.

Three limitations qualify this contribution. First, the proposed three-loop model is a theoretical synthesis that requires independent empirical validation before its interactions can be generalized beyond this synthesis. Second, the evidence base is concentrated in North American and European healthcare settings, with limited representation elsewhere. Third, quality appraisal relied on a single instrument, the MMAT, applied across heterogeneous study designs.

The most consequential direction for future research is empirical testing of whether interventions addressing one loop measurably affect the others, together with cross-context replication in low- and middle-income countries and culturally different healthcare settings. Validating the proposed loop structure against empirical work published after the 2015–2025 search window would complement this agenda. Organizations that recognize and systematically address this integrated barrier model may achieve the transformational improvements that Lean methodology promises.

## Figures and Tables

**Table 1:** Comparative mapping of baseline and synthesized literature for research gap identification.

Authors	Year of Research	Title	The Focus of the Study	Findings Related to Sustainability or Measurement
Kunnen, Y. S., Roemeling, O. P., & Smailhodzic, E.	2023	What are barriers and facilitators in sustaining lean management in healthcare? A qualitative literature review [16]	Identifies facilitators and barriers to sustaining Lean in healthcare, providing a conceptual framework.	The research highlights the importance of engaging the workforce to embed Lean into organizational culture, and creating learning and improvement cultures. A conceptual framework is developed to visualize relationships between barriers and facilitators.
Ellis, L. D. (based on Santos, K.)	2020	The Importance of Adopting a Lean Mindset and Culture for Health Care Organizations [20]	Describes the importance of a Lean mindset and culture in healthcare, outlining Lean principles and benefits.	This is a popular press article rather than an empirical study. Focuses on promoting Lean, not analyzing reasons for failed sustained adoption.
Melo, C., Berssaneti, F., Rampini, G., & Martinez, I.	2022	Exploring Barriers and Facilitators to Lean Implementation in Healthcare Organizations [17]	Explores barriers and facilitators to Lean implementation in healthcare, including Lean tools and practices.	The study identifies key barriers such as resistance to change, low staff involvement and facilitators such as leadership support and staff training. It also notes the importance of tools. It also indicates how Lean implementation leads to positive social and financial performance and value chain of service results.
Hung, D., Martinez, M., Yakir, M., & Gray, C.	2015	Implementing a Lean Management System in Primary Care: Facilitators and Barriers From the Front Lines [18]	Identifies key facilitators and barriers to implementing Lean in primary care through a case study.	The study emphasizes the complexity of Lean implementation and the importance of understanding early facilitators and barriers for maximizing its potential in healthcare delivery. The research highlights the need for staff empowerment and a culture of collaboration.

**Table 2:** Framework performance summary. Own elaboration.

Framework	Strengths	Weaknesses
HLA	Offers a clear framework for Lean maturity progression and emphasizes the importance of monitoring and continuous improvement [28].	Limited information on specific assessment criteria and methodology may not capture the nuances of Lean implementation in diverse healthcare settings; limited data on usage statistics and popularity [33].
LHISI	Captures frontline staff perspectives, provides a holistic view of Lean implementation across different units, relatively easy to administer and interpret [30].	It may be susceptible to response bias and limited data on usage statistics and popularity [33].
NSL	It provides insights into the impact of Lean on hospital-wide performance and uses robust statistical analysis [34].	It relies on self-reported data, which may be subject to bias and may not capture the nuances of Lean implementation in different units.

**Table 3:** Frameworks of sustainability. Own elaboration based on [28,29,34].

Framework	Focus	Sustainability
HLA	The overall maturity of Lean implementation	By emphasizing continuous improvement and monitoring of Lean implementation, the HLA framework promotes a culture of ongoing assessment and adaptation, which is crucial for sustaining Lean practices over time.
LHISI	Progress of Lean implementation	LHISI promotes sustainability by focusing on key Lean principles and their implementation progress. This allows organizations to track their Lean journey and identify areas for ongoing improvement, ensuring that Lean practices are embedded in their operations.
NSL	Impact of Lean adoption on hospital-wide performance	The NSL framework promotes sustainability by examining the impact of lean on hospital-wide performance. This helps organizations understand the long-term benefits of Lean adoption, encouraging them to sustain their efforts and continuously improve their processes.

**Table 4:** Factors influencing lean adoption derived from the included studies. Own elaboration based on the qualitative synthesis of the included literature (e.g., [14,16,25,31,38]).

Factor Category	Specific Factors	Influence the Adoption
Sociopolitical and external influence	Government policies and regulations, social networks, external environment	Can create a supportive or hindering environment for Lean adoption. For example, government incentives for quality improvement or collaborations with other healthcare organizations can facilitate Lean adoption.
Organizational characteristics	Leadership support, organizational culture, resources, absorptive capacity	Strong leadership commitment, a culture of continuous improvement, and adequate resources are crucial for successful Lean adoption.
Innovation characteristics	Complexity, relative advantage, compatibility, cost-efficacy, trialability	The perceived benefits, ease of implementation, and compatibility of Lean methodologies with existing practices can influence adoption decisions.
Staff/individual characteristics	Attitudes towards change, motivation, knowledge and skills, personal characteristics	Staff buy-in, motivation to improve processes, and adequate training are essential for successful Lean adoption.

**Table 5:** Approaches to measuring lean adoption. Own elaboration.

Approach	Methodology	Lean Adoption Is Understood as	Metrics Related to the Level of Adoption	Challenges in Measuring Sustained Adoption
Qualitative	Interviews, focus groups, content analysis	An organizational culture and philosophy of continuous improvement, a management system	Leadership engagement in daily routines, use of Lean tools in daily routines, commitment at different organizational levels, presence of evaluation and continuous improvement, extent of standardized work, usage of standardized meetings and follow-up metrics, use of digital daily management boards, staff ability to move around as needed	Misunderstanding of Lean philosophy by leaders, lack of staff commitment, resistance to change, complexity of implementation, difficulty measuring the impact of culture, lack of alignment with leadership, time constraints, difficulties in data collection, focus on individual processes rather than systemic change, difficulties maintaining long-term focus [14,25,38]
Quantitative	Surveys, statistical analysis	The degree to which Lean is diffused within hospital units, the timing of implementation, and self-reported maturity	Number of years doing Lean, number of units using Lean, self-reported maturity in using Lean, level of daily management system (DMS) implementation, staff involvement index, education and training index	Reliance on self-reported data, potential for instrument bias, difficulty in linking Lean to specific outcomes, difficulty in generalizing results [13,25,31]
Mixed methods	Combination of qualitative and quantitative approaches	A combination of factors, including adoption, implementation, outcomes, and cultural impact on Lean implementation	Number of ongoing Lean projects, staff familiarity with Lean tools, timing of the first Lean project, presence of an internal Lean expert, number of Lean projects, and level of Lean integration	Lack of standardized metrics, difficulty in isolating the impact of Lean from other factors, cultural differences influence implementation, and some measures are difficult to quantify [42,44,45]

**Table 6:** Methodological quality evaluation of the ten included studies using the Mixed Methods Appraisal Tool (MMAT), version 2018.

Study ID	First Author (Year)	Country	MMAT Category	S1	S2	Q.1	Q.2	Q.3	Q.4	Q.5	Overall (Y/CT/N)
IN-01	Kunnen (2023) [16]	International	Cat 1—Qual	Y	Y	Y	Y	Y	Y	Y	5/0/0
IN-02	Hirvelä (2024) [25]	Finland	Cat 1—Qual	Y	Y	Y	Y	Y	Y	Y	5/0/0
IN-03	Marsilio (2022) [31]	USA, Italy	Cat 4—QuantD	Y	Y	Y	CT	Y	CT	Y	3/2/0
IN-04	Rosa (2023) [14]	Italy	Cat 1—Qual	Y	Y	Y	Y	Y	Y	Y	5/0/0
IN-05	Sales Coll (2024) [44]	Spain	Cat 4—QuantD	Y	Y	Y	CT	Y	CT	Y	3/2/0
IN-06	Kabirinaeini (2023) [38]	Iran	Cat 1—Qual	Y	Y	Y	Y	Y	CT	Y	4/1/0
IN-07	Prado-Prado (2020) [45]	Spain	Cat 5—Mixed	Y	Y	Y	Y	CT	CT	CT	2/3/0
IN-08	Zdęba-Mozoła (2022) [46]	Poland	Cat 4—QuantD	Y	Y	Y	CT	Y	CT	CT	2/3/0
IN-09	Peimbert-García (2021) [42]	Finland, Mexico	Cat 4—QuantD	Y	Y	CT	CT	Y	CT	Y	2/3/0
IN-10	Tierney (2022) [13]	USA	Cat 3—QuantNR	Y	Y	CT	Y	Y	Y	Y	4/1/0

Abbreviations: Cat—MMAT design category; Qual—qualitative; QuantD—quantitative descriptive; QuantNR—quantitative non-randomized; Mixed—mixed methods; Y—Yes; CT—Can’t tell; N—No; S1, S2—MMAT screening questions (S1: clear research questions; S2: collected data allow addressing the research questions); Q.1–Q.5—design-specific MMAT criteria [43] (see Methods section and MMAT 2018 user guide for the full criterion text applicable to each category).

**Table 7:** Mapping of included studies to the three proposed loops.

Loop	Included Studies Cited as Direct Evidence in Discussion	Nature of Contribution
Measurement–Accountability Gap	IN-02 [25] Hirvelä; IN-03 [31] Marsilio; IN-10 [13] Tierney; IN-05 [44] Sales Coll; IN-08 [46] Zdęba-Mozoła; IN-09 [42] Peimbert-García	Cross-sectional (non-longitudinal) application of assessment frameworks; quality-improvement management processes; heterogeneity of measurement foci (economic impact, waste analysis, national-culture linkages), illustrating the lack of standardized longitudinal metrics
Tools–Philosophy Disconnect	IN-02 [25] Hirvelä (also above); IN-04 [14] Rosa; IN-07 [45] Prado-Prado	Recurring divergence between discrete tool deployment and underlying continuous-improvement philosophy across Finland, Italy, and Spain
Complexity–Resistance Cycle	IN-01 [16] Kunnen; IN-06 [38] Kabirinaeini	Organizational complexity and staff/clinician resistance dynamics catalyzed within autonomous clinical workflows

## Data Availability

No new data were created or analyzed in this study. Data sharing is not applicable to this article.

## Supplementary Materials

The following supporting information can be downloaded at: https://www.mdpi.com/article/10.3390/healthcare14152403/s1.

## Abbreviations

LM	Lean Management
HLA	Healthcare Lean Assessment
LHISI	Lean Healthcare Implementation Self-Assessment Instrument
NSL	National Survey of Lean
PRISMA	Preferred Reporting Items for Systematic Reviews and Meta-Analyses
GDP	Gross Domestic Product
