# Supplementary material for: Lean Adoption in Healthcare: Between Promise and Practice
Source: Healthcare (Basel). 2026 Aug 5;14(15):2403. doi: 10.3390/healthcare14152403 (PMC13463896; doi:10.3390/healthcare14152403)
Supplement: Supplementary file 1 [file healthcare-14-02403-s001.zip › healthcare-4375077-supplementary.pdf]

Supplementary material S1: PRISMA 2020 compliant search protocol Systematic Literature Review

SECTION S1

SEARCH STRATEGY – BOOLEAN QUERIES BY DATABASE
The search strategy employed a combination of topic-related keywords and setting-related keywords utilizing Boolean operators tailored to each database's syntax. The search was conducted in November 2025.

S1.1 SCOPUS (https://www.scopus.com)

Records retrieved: 198
Principal search string: TITLE-ABS-KEY(("lean adoption" OR "lean adoption rate" OR "lean implementation" OR "lean sustainability") AND ("health care" OR "healthcare" OR "hospital")) AND (LIMIT-TO(PUBYEAR, 2015) OR LIMIT-TO(PUBYEAR, 2016) OR LIMIT-TO(PUBYEAR, 2017) OR LIMIT-TO(PUBYEAR, 2018) OR LIMIT-TO(PUBYEAR, 2019) OR LIMIT-TO(PUBYEAR, 2020) OR LIMIT-TO(PUBYEAR, 2021) OR LIMIT-TO(PUBYEAR, 2022) OR LIMIT-TO(PUBYEAR, 2023) OR LIMIT-TO(PUBYEAR, 2024) OR LIMIT-TO(PUBYEAR, 2025)) AND (LIMIT-TO(DOCTYPE, "ar") OR LIMIT-TO(DOCTYPE, "re")) AND (LIMIT-TO(LANGUAGE, "English"))

Search fields: Title, Abstract, Keywords
Filters: Document type (Article or Review), Language (English), Publication years 2015-2025

S1.2 EMERALD INSIGHT (https://www.emerald.com/insight)

Records retrieved: 47
Search string: All: (("lean adoption" OR "lean adoption rate" OR "lean implementation" OR "lean sustainability") AND ("health care" OR "healthcare" OR "hospital")) | Publication Date:[2015 TO 2025] | Content Type: Journal Article | Language: English
Search fields: All fields (full text) Filters: Publication date 2015-2025, Content type (Journal Article), Language (English)

S1.3 PUBMED / MEDLINE (https://pubmed.ncbi.nlm.nih.gov)

Records retrieved: 64
Search string: (("lean adoption"[Title/Abstract] OR "lean adoption rate"[Title/Abstract] OR "lean implementation"[Title/Abstract] OR "lean sustainability"[Title/Abstract]) AND ("health care"[Title/Abstract] OR "healthcare"[Title/Abstract] OR "hospital"[Title/Abstract])) AND ("2015"[Date - Publication] : "2025"[Date - Publication]) AND (English[lang])

Search fields: Title/Abstract Filters: Publication date 2015-2025, Language (English)

S1.4 GOOGLE SCHOLAR (https://scholar.google.com)

Note: Used as a supplementary search source to capture gray literature and mitigate publication bias. Specifically, Google Scholar serves as a coverage safeguard to retrieve papers inconsistently indexed elsewhere; all identified records remain subject to the same peer-review eligibility criteria as primary databases.

Records retrieved: 2,845
Search string: "lean adoption" OR "lean adoption rate" OR "lean implementation" OR "lean sustainability" AND "health care" OR "healthcare" OR "hospital"
Additional filters applied via interface:

- Custom range: 2015-2025
- Language: English (default)
- Include citations: No

SECTION S2 - PRISMA 2020 FLOW DIAGRAM

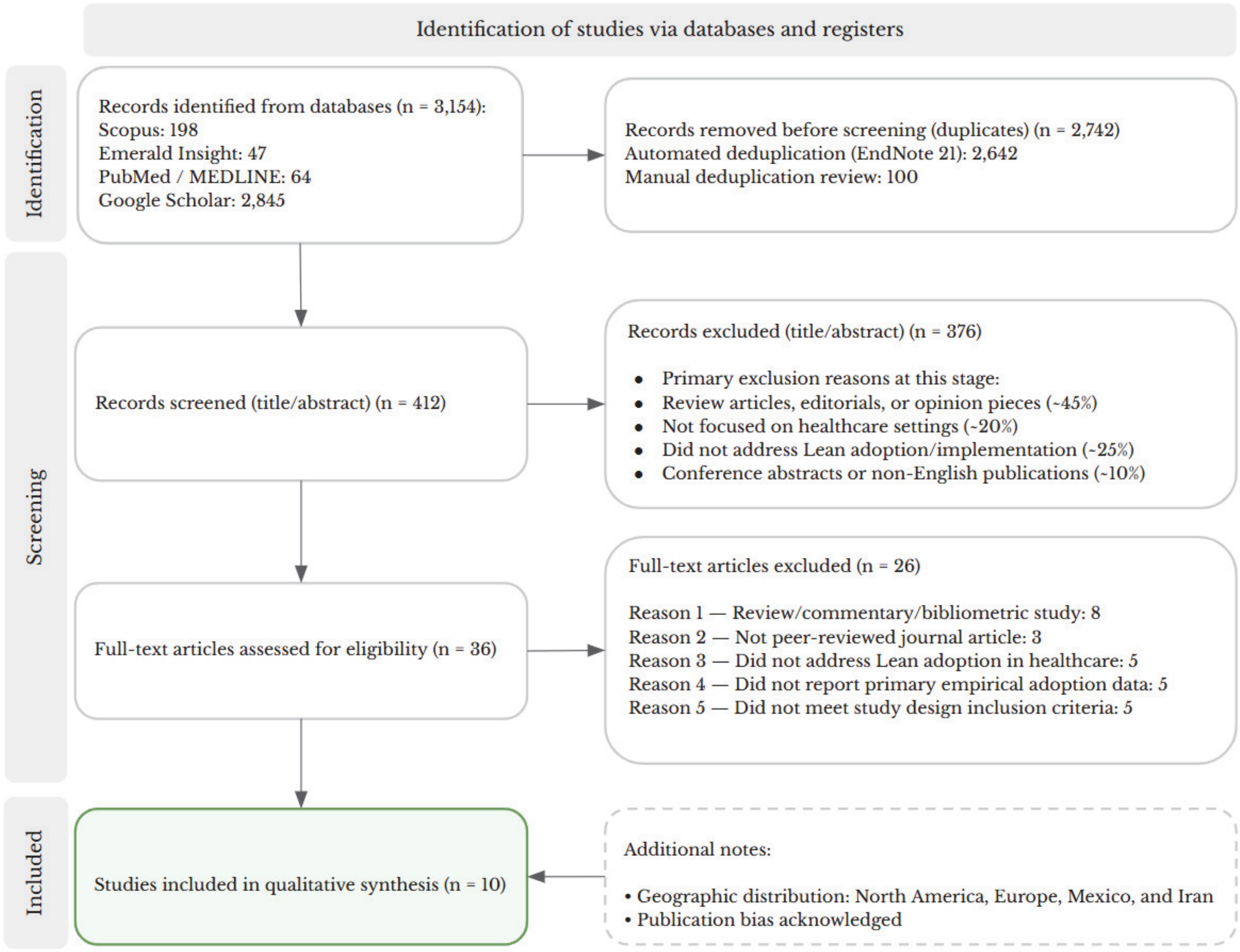

SECTION S3: INCLUSION AND EXCLUSION CRITERIA

INCLUSION CRITERIA:

1. Peer-reviewed articles published in English between 2015 and 2025
2. Studies focused on healthcare settings (hospitals, clinics, primary care, or integrated health systems)
3. Studies addressing Lean Management adoption, implementation, or sustainability as a primary focus
4. Studies employing qualitative, quantitative, or mixed-methods research designs
5. Studies reporting primary empirical data (adoption rates, measurement frameworks, barriers/facilitators)

EXCLUSION CRITERIA:

1. Non-peer-reviewed articles (popular press, blog posts, conference proceedings without journal publication)
2. Studies not focused on healthcare settings (manufacturing, automotive, general services)
3. Studies not directly addressing LM adoption or long-term implementation (focus exclusively on financial outcomes, supply chain, environmental sustainability, or Industry 4.0)
4. Review articles, editorials, opinion pieces, or bibliometric studies without primary empirical data
5. Studies published outside the 2015-2025 date range
6. Studies not published in English

SECTION S4: EXCLUDED STUDIES (n = 26) — FULL-TEXT ASSESSMENT

The following 26 articles passed title/abstract screening but were excluded during full-text assessment. Each entry includes the exclusion reason aligned with the predefined criteria.

CATEGORY S1

Review, commentary, or bibliometric study (no primary empirical data) — 8 studies

EX-04 | Thomas A, Suresh M (2024) Title: Readiness of lean sustainability in healthcare organizations Journal: Int J Pharmaceutical and Healthcare Management DOI: 10.1108/IJPHM-07-2022-0064 Reason: Systematic literature review; synthesizes existing research without reporting primary empirical data on Lean adoption rates.

EX-05 | Leite H, Bateman N, Radnor Z (2020) Title: Beyond the ostensible: an exploration of barriers to lean implementation and sustainability in healthcare Journal: Production Planning & Control DOI: 10.1080/09537287.2019.1623426 Reason: Systematic literature review; secondary analysis synthesizing existing barriers without primary adoption measurement data.

EX-06 | Wagner RS, Hattingh TS, Meijer H (2022) Title: Factors affecting the sustainability of lean in healthcare: a systematic literature review Journal: South African Journal of Industrial Engineering DOI: 10.7166/33-3-2813 Reason: Systematic literature review; secondary analysis of sustainability factors without primary empirical adoption data.

EX-07 | Flynn R, Newton AS, Rotter T, Hartfield D, Walton S, Fiander M, Scott SD (2018) Title: The sustainability of Lean in pediatric healthcare: a realist review Journal: Systematic Reviews DOI: 10.1186/s13643-018-0800-z Reason: Realist review methodology; synthesizes theoretical contexts and mechanisms without reporting primary empirical adoption rates.

EX-10 | Lima RM, Dinis-Carvalho J, Souza TA, et al. (2021) Title: Implementation of lean in health care environments: an update of systematic reviews Journal: International Journal of Productivity and Performance Management DOI: 10.1108/IJLSS-07-2019-0074 Reason: Update of systematic reviews; secondary analysis aggregating prior review findings without primary empirical data.

EX-19 | D'Andreamatteo A, Ianni L, Lega F, Sargiacomo M (2015) Title: Lean in healthcare: a comprehensive review Journal: Health Policy DOI: 10.1016/j.healthpol.2015.02.002 Reason: Comprehensive review article; no primary empirical data on Lean adoption measurement or rates.

EX-25 | Kaltenbrunner M, Mathiassen SE, Bengtsson L (2017) Title: A questionnaire measuring staff perceptions of Lean adoption in healthcare: commentary Journal: BMC Health Services Research DOI: 10.1186/s12913-017-2163-x (commentary) Reason: Commentary/response article; not a primary empirical study reporting adoption measurement or rates.

EX-26 | Nicholas J (2023) Title: Lean daily management in healthcare: origins, practices, and associations with lean leadership and lean sustainability Journal: Total Quality Management & Business Excellence, DOI: 10.1080/14783363.2023.2182677 Reason: Systematic/exploratory literature review; synthesizes evidence on daily management practices in healthcare without primary empirical adoption data.

CATEGORY S2

Not published in a peer-reviewed academic journal — 3 studies

EX-01 | Ellis LD (2020) Title: The Importance of Adopting a Lean Mindset and Culture for Health Care Organizations Source: Harvard Medical School Postgraduate Education (online) DOI: N/A (popular press article) Reason: Popular press article from Harvard Medical School; not a peer-reviewed empirical study.

EX-02 | Melo C, Berssaneti F, Rampini G, Martinez I (2022) Title: Exploring Barriers and Facilitators to Lean Implementation in Healthcare Organizations Source: Proceedings of the International Conference on Industrial Engineering and Operations Management (IEOM), Rome DOI: N/A Reason: Conference proceedings; not a peer-reviewed journal article

EX-09 | Manguinuri-Sanchez RB, Acuña-Chávez JJ, Quiroz-Flores JC (2025) Title: Lean Healthcare Model for SMEs: Improving Efficiency and Sustainability in Small and Medium-Sized Health Services Source: Proceedings of the 2nd World Congress on Industrial Engineering and Operations Management (IEOM), Windsor, Canada, October 2025 https://index.ieomsociety.org/index.cfm/item/56416 Reason: Conference proceedings; not a peer-reviewed journal article.

CATEGORY S3

Did not address Lean adoption, implementation, or sustainability in healthcare — 5 studies

EX-08 | Borges GA, Tortorella G, Rossini M, Portioli-Staudacher A (2019) Title: Lean implementation in healthcare supply chain: a scoping review Journal: Journal of Health Organization and Management DOI: 10.1108/JHOM-06-2018-0176 Reason: Scoping review focused on healthcare supply chain logistics; does not address Lean adoption in clinical/patient care settings.

EX-12 | Patri R, Suresh M (2018) Title: Factors influencing lean implementation in healthcare organizations: an ISM approach Journal: International Journal of Healthcare Management DOI: 10.1080/20479700.2017.1300380 Reason: Theoretical ISM modeling study; develops a hierarchical factor model without empirical adoption rate measurement.

EX-13 | Ilangakoon TS, Weerabahu SK, et al. (2022). Title: Adoption of Industry 4.0 and lean concepts in hospitals for healthcare operational performance improvement. Journal: International Journal of Productivity and Performance Management DOI: 10.1108/IJPPM-12-2020-0654 Reason: Focuses on combined Industry 4.0 and Lean adoption; not directly addressing Lean management adoption specifically.

EX-17 | Narayanan S, Vickery SK, Nicolae ML, et al. (2022) Title: The effects of lean implementation on hospital financial performance Journal: Decision Sciences DOI: 10.1111/deci.12510 Reason: Focuses exclusively on financial performance outcomes; does not address Lean adoption rates or sustainability factors.

EX-22 | Singh P, Singh A, Singh P (2019) Title: Lean in healthcare organization: an opportunity for environmental sustainability Journal: Benchmarking: An International Journal DOI: 10.1108/BIJ-04-2018-0104 Reason: Focuses on environmental sustainability outcomes of Lean; not directly addressing Lean adoption measurement or barriers

---

CATEGORY S4

Did not report primary empirical data on Lean adoption measurement — 5 studies

EX-11 | Hallam CRA, Contreras C (2018) Title: Lean healthcare: scale, scope and sustainability Journal: International Journal of Health Care Quality Assurance DOI: 10.1108/IJHCQA-02-2017-0023 Reason: Conceptual analysis of Lean healthcare implementation; does not present primary empirical adoption measurement data.

EX-14 | Akmal A, Foote J, Greatbanks R, Kaur M (2022) Title: Understanding resistance in lean implementation in healthcare environments: an institutional logics perspective Journal: Production Planning & Control DOI: 10.1080/09537287.2020.1823510 Reason: Theoretical perspective paper using institutional logics; does not measure or report Lean adoption rates.

EX-15 | Bengtsson L, Mathiassen SE (2017) Title: A questionnaire measuring staff perceptions of Lean adoption in healthcare: development and psychometric testing Journal: BMC Health Services Research DOI: 10.1186/s12913-017-2163-x Reason: Instrument development and validation study; focuses on psychometric testing rather than reporting adoption rates.

EX-16 | Dorval M, Jobin MH (2022) Title: A conceptual model of Lean culture adoption in healthcare Journal: International Journal of Productivity and Performance Management DOI: 10.1108/IJPPM-06-2020-0345 Reason: Conceptual/theoretical model development; no primary empirical data collection or adoption measurement.

EX-20 | Nawanir G, Fauzi MA (2025) Title: Lean healthcare: a science mapping of current progress and future research avenues Journal: Journal of Health Organization and Management DOI: DOI: 10.1108/JHOM-04-2024-0167 Reason: Bibliometric/science mapping study; analyzes publication trends rather than reporting empirical Lean adoption data.

CATEGORY S5

Did not meet inclusion criteria for study design or scope — 5 studies

EX-03 | Hung D, Martinez M, Yakir M, Gray C (2015) Title: Implementing a Lean Management System in Primary Care: Facilitators and Barriers From the Front Lines Journal: Quality Management in Health Care DOI: 10.1097/QMH.0000000000000062 | PubMed PMID: 26115057 Reason: Single-setting qualitative case study; limited focus on adoption measurement; narrow scope (one primary care clinic).

EX-18 | Harrison MI, Paez K, Carman KL, et al. (2016). Title: Effects of organizational context on Lean implementation in five hospital systems. Journal: Health Care Management Review DOI: 10.1097/HMR.0000000000000049 Reason: Multi-case study examining implementation process; does not report standardized adoption rates or frameworks.

EX-21 | Régis TKO, Santos LC, Gohr CF (2019) Title: A case-based methodology for lean implementation in hospital operations Journal: Journal of Health Organization and Management, DOI: 10.1108/JHOM-09-2018-0267 | PubMed PMID: 31625821 | URL: https://pubmed.ncbi.nlm.nih.gov/31625821/
Reason: Methodology-focused paper; does not report adoption rates, measurement frameworks, or sustainability outcomes.

EX-23 | Noori B (2015) Title: The critical success factors for successful lean implementation in hospitals Journal: International Journal of Productivity and Quality Management (IJPQM) DOI: 10.1504/IJPQM.2015.065987 | URL: https://www.inderscienceonline.com/doi/abs/10.1504/IJPQM.2015.065987 Reason: Theoretical framework development on critical success factors; does not report empirical adoption rates.

EX-24 | Fournier PL, Lapointe L, Momani A (2021) Title: Lean implementation in healthcare: offsetting physicians' resistance to change Journal: Production Planning & Control DOI: 10.1080/09537287.2021.1938730 Reason: Focuses exclusively on physician resistance mechanisms; does not measure or report Lean adoption data.

SECTION S5 - INCLUDED STUDIES (n = 10)

The following 10 studies met all inclusion criteria and were included in the qualitative synthesis:

IN-01 | Kunnen YS, Roemeling OP, Smailhodzic E (2023) Title: What are barriers and facilitators in sustaining lean management in healthcare? A qualitative literature review Journal: BMC Health Services Research DOI: 10.1186/s12913-023-09978-4 Type: Qualitative review Country: International

IN-02 | Hirvela I, Torkki P, Javanainen M, Reponen E (2024) Title: The maturity of lean management in a large academic medical center in Finland: a qualitative study Journal: International Journal for Quality in Health Care DOI: 10.1093/intqhc/mzae111 Type: Qualitative study Country: Finland

IN-03 | Marsilio M, Pisarra M, Rubio K, Shortell S (2022) Title: Lean adoption, implementation, and outcomes in public hospitals: benchmarking the US and Italy health systems Journal: BMC Health Services Research DOI: 10.1186/s12913-022-07473-w Type: Quantitative survey Country: USA, Italy

IN-04 | Rosa A, Marolla G, McDermott O (2023) Title: A cross-organizational Lean deployment in an Italian regional healthcare system Journal: International Journal of Health Care Quality Assurance DOI: 10.1108/IJHCQA-06-2023-0045 Type: Qualitative study Country: Italy

IN-05 | Sales Coll M, De Castro R, Ochoa De Echaguen A, Martinez Ibanez V (2024) Title: Economic Impact of Lean Healthcare Implementation on the Surgical Process Journal: Healthcare (MDPI) DOI: 10.3390/healthcare12050512 Type: Quantitative Country: Spain

IN-06 | Kabirinaeini M, Jamali H, Elahi Z (2023) Title: A Framework for Implementing Lean Management in the Hospital Emergency Department Journal: Trends in Medical Sciences DOI: 10.5812/tms-138624 Type: Framework development Country: Iran

IN-07 | Prado-Prado JC, Garcia-Arca J, Fernandez-Gonzalez AJ, Mosteiron-Anon M (2020) Title: Increasing Competitiveness through the Implementation of Lean Management in Healthcare Journal: International Journal of Environmental Research and Public Health DOI: 10.3390/ijerph17144981 Type: Mixed methods Country: Spain

IN-08 | Zdeba-Mozola A, Rybarczyk-Szwajkowska A, Czapla T, Marczak M, Kozlowski R (2022) Title: Implementation of Lean Management in a Multi-Specialist Hospital in Poland and the Analysis of Waste Journal: International Journal of Environmental Research and Public Health DOI: 10.3390/ijerph19020800 Type: Case study Country: Poland

IN-09 | Peimbert-Garcia RE, Jorma T, Cardenas-Barrón LE, Nucamendi-Guillén SM, Garcia-Reyes H (2021) Title: Linking Lean Adoption and Implementation in Healthcare to National Cultures Journal: Sustainability (MDPI) DOI: 10.3390/su13168855 Type: Quantitative Country: International (Finland, Mexico)

IN-10 | Tierney AA, Shortell SM, Rundall TG, Blodgett JC, Reponen E (2022) Title: Examining the Relationship Between the Lean Management System and Quality Improvement Care Management Processes Journal: Quality Management in Health Care DOI: 10.1097/QMH.0000000000000318 Type: Quantitative Country: USA
